# Ewe-lamb bond of experienced and inexperienced mothers undernourished during gestation

**DOI:** 10.1038/s41598-021-84334-2

**Published:** 2021-02-25

**Authors:** Aline Freitas-de-Melo, Raquel Pérez-Clariget, Angélica Terrazas, Rodolfo Ungerfeld

**Affiliations:** 1 Departamento de Biociencias Veterinarias, Universidad de la República, Lasplaces 1620, 11600 Montevideo, Uruguay; 2 Departamento de Producción Animal y Pasturas, Facultad de Agronomía, Universidad de la República, Garzón 780, 12 400 Montevideo, Uruguay; 3 Departamento de Ciencias Pecuarias, Facultad de Estudios Superiores Cuautitlán, Universidad Nacional Autónoma de México, km 2.5 Cuautitlán-Teoloyucan San Sebastián Xhala, 547114 Cuautitlán Izcalli, Edo. México México

**Keywords:** Developmental biology, Zoology

## Abstract

The aims were to compare ewe-lamb behaviours between primiparous (PRI) and multiparous (MUL) undernourished grazing ewes at birth and at 3 months of age, and to determine if mothers’ parity affects milk yield and composition, and lambs’ body weight (BW). Food availability restricted the nutritional requirements from day 30 to day 143 of gestation. The MUL ewes had greater BW than the PRI during gestation, and their lambs tended to vocalize less frequently until their first suckle. PRI ewes both displayed a lower frequency of acceptance behaviours and, a greater number of high-pitched bleats toward the alien lamb than toward that of their own, but MUL did not. PRI ewes produced less milk than the MUL ewes. The heart rate was greater in lambs reared by MUL ewes than by PRI. Although PRI ewes had a lower BW during gestation, this difference was stable throughout and did not affect the establishment of the ewe-lamb bond neither at birth nor at 3 months postpartum. At least under nutritionally restricted conditions during gestation, inexperienced mothers appeared to have had a shorter sensitivity period of maternal responsiveness than that of experienced mothers.

## Introduction

Ewes establish a selective bond with their offspring after birth, during a sensitive period of maternal responsiveness^1^. Accordingly, ewes only display maternal care toward the offspring, whose odour was identified during that period, thus rejecting lambs not of their own. During the first weeks of life, the mother is the main source of food for the lamb, providing heat together with protection against predators, increasing the probability of lambs’ survival^2^. As the postpartum period advances, nursing becomes less frequent^3^ as the lamb gradually gains nutritional and social independence^4^.

Many factors, including that of parity, breed and maternal nutrition, influence the establishment and the evolution of ewe-lamb bond^5,6^. For instance, in extensive grazing sheep systems based on natural pastures, pregnant ewes undergo periods of undernutrition as gestation coincides with winter, during which time the lowest natural pastures quantity together with the poorest quality, are observed. Undernutrition affects the subsequent ewe-lamb relationship at birth and during postpartum period^7,8^. However, the effect of undernutrition might be more greatly realised in multiparous (MUL) than in primiparous (PRI) ewes, as MUL ewes produce heavier lambs^9^. Thus, food restriction might impose greater limitations on foetus growth and lambs from MUL ewes, affecting the ewe-lamb behaviours at birth and the independence from the dam during lactation. In sheep production systems, where pregnant ewes are well fed, PRI ewes lick their lambs for the first time generally at a later stage, spend less time licking and, make more movements when lambs attempt to suckle than MUL ewes^10,11^. Additionally, while MUL ewes prefer their lamb over an alien lamb 6 h after birth, PRI ewes do not^12^. Lambs born from PRI mothers are lighter and thus, slower to stand up and suckle for the first time than lambs born from MUL mothers^9^. Even one day after birth, lambs born from PRI ewes have impaired ability to distinguish their mother from that of an alien one^13^, which is related with a greater mortality rate of their lambs^14^. However, to the best of our knowledge, there is paucity on information on the evolution of the ewe-lamb bond throughout lactation according to the parity. In particular, PRI ewes and their lambs display a more intensive physical motion in a partial separation test than MUL ewes and their lambs at 2 months of age^15^. This might be a consequence of the greater reactivity of PRI ewes to a challenge situation^16^. Parity also affects the bond between the mother and its offspring in cattle, as MUL cows vocalize and pace more after early-weaning than PRI cows^17^.

The aim of this study was to compare ewe-lamb behaviours between PRI and MUL undernourished grazing ewes at birth and at 3 months of age. It has been demonstrated that PRI ewes produce less milk^18^, and therefore, their lambs grow at a slower rate than those from MUL ewes^19^, which may very well affect their nutritional independence from their mother and the strength of bond during lactation. A complementary aim was to determine if milk yield and composition, together with lambs’ weight, differ according to their mothers’ parity.

## Results

### Body weight, BCS and udder size of ewes during gestation

**Figure 1:**
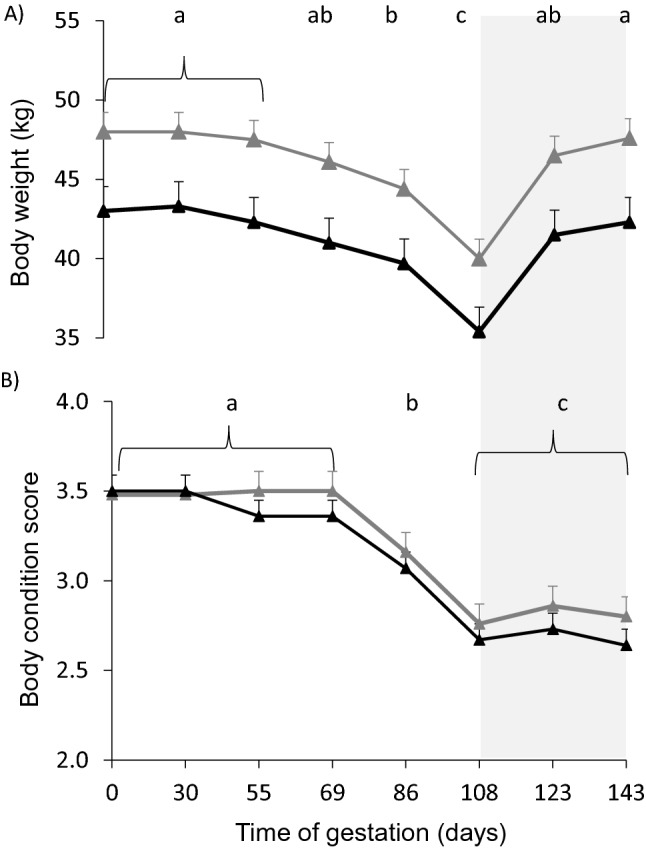
Body weight (**A**) and body condition score (**B**) of multiparous (gray filled triangle) and primiparous (black filled triangle) ewes. Body weight was greater and body condition score tended to be greater in multiparous than in primiparous ewes (*p* < 0.0001 and *p* = 0.07, respectively). Different letters indicate significant differences between gestation days (*P* < 0.0001). The light grey area represents the period when all ewes were supplemented with 300 g/animal/day of rice bran.

The MUL ewes were heavier and tended to have a greater BCS than PRI ewes during gestation (BW: 46.0 ± 0.4 kg vs 41.1 ± 0.5 kg; DF = 261; F = 51.73; *p* < 0.0001; BCS: 3.2 ± 0.04 vs 3.1 ± 0.04, respectively; DF = 261; F = 3.35; *p* = 0.07). The BW and BCS of ewes changed with time (*p* < 0.0001). From day 86 to day 108 of gestation, BW decreased (*p* < 0.002); and from day 108 to day 123 of gestation, it increased (*p* < 0.0001) (Fig. 1A and B). From day 69 to day 108 of gestation BCS decreased in both groups (*p* < 0.0001), remaining stable from day 108 to day 143 of gestation. There was no interaction between group and time in BW and BCS.

The volume of the udder did not differ between MUL and PRI ewes on day 143 of gestation (936.03 ± 103.4 cm^3^ vs 794.9 ± 116.9 cm^3^, respectively).

### Serum biochemical variables

**Figure 2:**
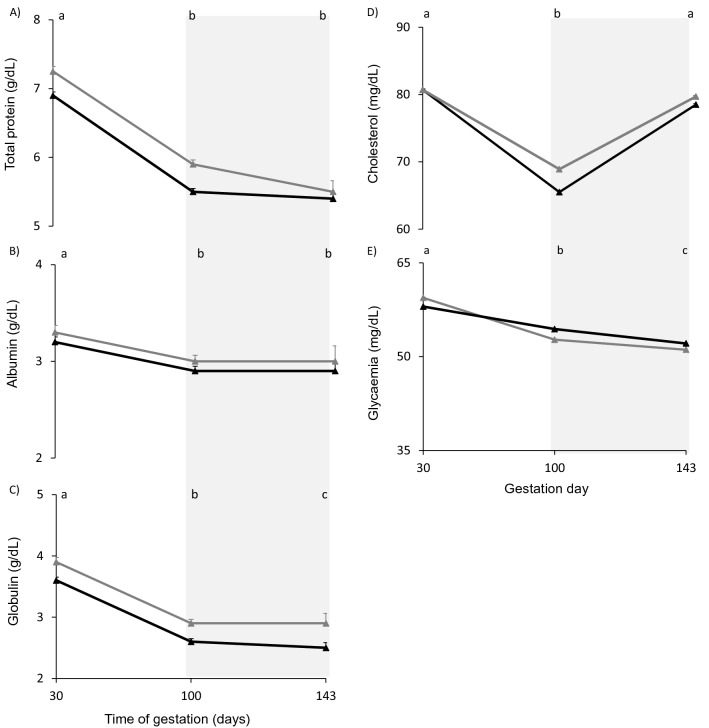
Total protein (**A**), albumin (**B**), globulin (**C**), cholesterol (**D**) and glucose (**E**) concentrations (LS mean ± s.e.m.) of multiparous (gray filled triangle) and primiparous (black filled triangle) ewes. Total protein and globulin concentrations were greater in multiparous than in primiparous ewes (*p* < 0.02). Different letters indicate significant differences between the days (*P* < 0.0009). The light grey area represents the period when all ewes were supplemented with 300 g/animal/day of rice bran.

The MUL ewes had greater concentrations of total serum protein (6.3 ± 0.06 g/dL vs 6.0 ± 0.07 g/dL; DF = 91; F = 6.38; *p* = 0.01) and globulins (3.1 ± 0.04 g/dL vs 2.9 ± 0.05 g/dL; DF = 90; F = 5.96; *p* = 0.02) than PRI ewes (Fig. 2A and C). There were no effects of parity in albumin, cholesterol or glucose concentrations (Fig. 2B, D and E). All the biochemical variables changed with days, decreasing from day 30 to day 100 of gestation (*p* < 0.007). There were no interactions between parity and time in any biochemical variable.

### Behaviours, body weight and body temperature at birth

Duration of the first licking, latency of the lamb to stand up, latency to first suckling, duration of first suckling and number of vocalizations of the mother and lamb until first suckling did not differ between groups (Table 1). The frequency of vocalizations of the lambs from birth until first suckling (number of vocalizations of the lamb until first suckling/the time required to suckle for the first time) tended to be lower in lambs born from MUL ewes than in the lambs born from PRI ewes (Table 1; *P* = 0.06). The parity did not affect lamb BW at birth, rectal and surface temperatures. Birth weight was 4.0 ± 0.1 kg vs 3.8 ± 0.2 kg for MUL and PRI ewes. Surface temperatures on the neck area were 24.1 ± 0.8 °C and 22.9 ± 0.8 °C in MUL (n = 12) and PRI (n = 8) ewes, respectively. Surface temperature on the hips area were 25.2 ± 1.0 °C and 24.7 ± 1.0 °C in MUL (n = 12) and PRI ewes (n = 8) respectively; and rectal temperature were 39.0 ± 0.7 °C and 37.9 ± 0.8 °C in MUL (n = 11) and PRI (n = 8), respectively.

**Table 1:** Behaviours [LS mean ± sem (min–max values)] of multiparous and primiparous ewes and their lambs recorded at birth.

	Multiparous	*N*	Primiparous	*N*	P
**Ewes’ behaviours**					
Duration of the first licking (min)	9.5 ± 2.5 (1–32)	15	9.9 ± 3.5 (1–35)	9	ns
Number of vocalizations until first suckling	73.9 ± 17.7 (0–207)	14	98.7 ± 25.2 (3–268)	10	ns
Vocalizations until first suckling (number/min)	1.5 ± 0.4 (0–4.4)	13	2.4 ± 0.7 (0.02–7.1)	9	ns
**Lambs’ behaviours**					
Time to first stand (min)	23.4 ± 3.7 (6–57)	15	23.4 ± 9.6 (6–101)	9	ns
Time to first suckling (min)	46.1 ± 8.1 (16–120)	14	60.7 ± 11.6 (31–120)	9	ns
Duration of first suckling (s)	87.6 ± 12.9 (15–180)	12	97.1 ± 17.1 (60–180)	7	ns
Number of vocalizations until first suckling	50.6 ± 14.0 (4–124)	13	83.4 ± 20.6 (13–169)	9	ns
Vocalizations until first suckling (number/min)	1.0 ± 0.3 (0.1–3.8)	13	1.9 ± 0.4 (0.4–4.4)	8	0.06

*ns* not significative (*P* > 0.1).

### Behaviours during the selectivity test

The results of this test are presented in Table 2. While PRI ewes emitted a lower number of low-pitched bleats toward the alien lamb (*p* = 0.04; W = 39.5) and allowed these lambs to stay near the udder for a shorter time than their own lamb (*p* = 0.04; W = 33.0), MUL ewes did not. However, both, MUL (*p* = 0.07) and PRI (*p* = 0.1) ewes tended to accept more frequently their own lamb that the alien lamb at the udder.

**Table 2:** Behaviours (mean ± sem) toward the alien or the mother’s lambs during a selectivity test carried out approximately two hours after lambing, in multiparous (n = 12) or primiparous (n = 9) ewes.

Behaviours	Multiparous		Primiparous	
	Own	Alien	Own	Alien
Number of low-pitched bleats	8.2 ± 1.6^a^	4.2 ± 1.2^a^	10.5 ± 3.0^a^	2.2 ± 0.8^b^
Number of high-pitched bleats	4.5 ± 2.8^a^	17.7 ± 5.2^a^	2.9 ± 1.0^a^	20.8 ± 3.7^b^
Time near the udder (s)	37.2 ± 12.7^a^	19.7 ± 14.0^a^	27.7 ± 10.3^a^	4.9 ± 2.3^b^
Number of udder rejection	0.6 ± 0.5^a^	2.3 ± 0.7^b^	0.1 ± 0.1^a^	1.0 ± 0.3^b^
Number of udder acceptance	1.7 ± 0.5^x^	0.4 ± 0.2^y^	1.7 ± 0.5^x^	0.4 ± 0.2^y^
Number of agressive behaviours	0.08 ± 0.08^a^	3.4 ± 1.2^b^	0.0 ± 0.0^a^	1.0 ± 0.3^b^

Means within the same row bearing different letters (a and b) differ significantly (*P* ≤ 0.04) or tented to differ (x and y; 0.07 ≤ *p* ≤ 0.1).

In relation to the rejecting behaviours, while PRI ewes emitted a greater number of high-pitched bleats toward the alien lambs than toward their own lamb (W = 39.0; *p* = 0.01), MUL ewes did not. The MUL (W = 45.0; *p* = 0.006) and PRI (W = 17.0; *p* = 0.02) ewes rejected more frequently the alien lamb than their lamb at the udder. Lastly, both MUL (W = 45.0; *p* = 0.006) and PRI (W = 21.0; *p* = 0.05) ewes displayed a greater number of aggressions toward the alien lamb than toward their own lambs.

### Body weight and BCS of ewes and lambs during the postpartum period

The MUL ewes tended to be heavier than PRI ewes also during this period (41.3 ± 0.8 kg vs 39.0 ± 0.1 kg; DF = 58; F = 2.94; *p* < 0.09), but BCS did not differ between groups (MUL: 2.5 ± 0.08 vs PRI: 2.7 ± 0.08). The BCS did not change with time, but BW of ewes decreased from day 45 (41.6 ± 1.0 kg) until day 91 (38.7 ± 1.0 kg; DF = 58; F = 4.29; *p* = 0.04), independently of the parity. There were no significant interactions between groups and time in ewes’ BW or BCS.

Lambs born from MUL ewes tended to be heavier than those born from PRI ewes (15.0 ± 0.4 kg vs 13.8 ± 0.5 kg; respectively; DF = 58; F = 3.57; *p* < 0.06). The BW of lambs increased from day 45 (12.3 ± 0.5 kg) until day 91 (16.5 ± 0.5 kg; DF = 58; F = 41.93; *p* < 0.0001) postpartum. There were no interactions between groups and time in BW and BCS of the lambs.

### Short-term maternal separation test

Parity did not affect any behaviour recorded during the test (Table 3) or the rectal temperature (MUL: 40.6 ± 0.07 °C vs PRI: 40.8 ± 0.09 °C). The heart rate was greater in lambs born from MUL than from PRI ewes (148.8 ± 3.1 beats/min vs 138.2 ± 3.9 beats/min; DF = 57; F = 4.57; *p* = 0.04, respectively), but heart rate did not change with days, and there was no interaction between group and days.

**Table 3:** Behaviours during the ewe-lamb short-term maternal separation test (LS mean ± sem) in lambs at 3 months of age reared by multiparous (n = 19) or primiparous (n = 12) ewes.

	Multiparous	Primiparous
**Behaviours while the lamb remained isolated**		
Time walking (s)	24.5 ± 4.4	37.2 ± 10.7
Number of vocalizations	35.2 ± 4.0	30.4 ± 5.0
**Behaviours after including the mother in the test**		
Time walking (s)	46.6 ± 7.9	48.7 ± 14.9
Number of vocalizations	30.2 ± 4.0	26.2 ± 5.0
Number of times that the lambs crossed the line	5.6 ± 0.9	5.7 ± 1.2
Time in the zone near fence (s)	203.5 ± 17.3	230.6 ± 19.2
Number of contact attempts	10.0 ± 2.5	10.7 ± 1.8

There were no significant differences between groups.

Independently of the parity, the lambs walked longer when their mother was in the other side of the fence than when they were alone (time walking: from 29.4 ± 0.6 s to 49.3 ± 0.4 s; DF = 58; F = 4.84; *p* = 0.03). The rectal temperature also increased after the second part of the test, from 40.5 ± 0.08 to 40.9 ± 0.08 °C (DF = 57; F = 7.42; *p* = 0.009).

### Milk yield and milk composition

MUL ewes produced more milk/day than the PRI ewes at 91 days postpartum (DF = 28; F = 4.59; *p* = 0.04), without differences in fat, protein and lactose milk content (Table 4). The total milk solid content tended to be greater in MUL than in PRI ewes (Table 4; *p* = 0.07).

**Table 4:** Milk composition and milk yield (LS mean ± sem) at 91 days postpartum in multiparous (n = 19) or primiparous (n = 12) ewes.

Milk yield and content	Multiparous	Primiparous	*P*
Milk yield (kg)	0.4 ± 0.03^b^	0.3 ± 0.04^a^	0.04
Fat milk (g)	29.9 ± 2.4	24.8 ± 3.2	ns
Protein (g)	19.7 ± 1.5	15.7 ± 2.1	ns
Lactose (g)	20.9 ± 1.7	16.7 ± 2.3	ns
Total solids (g)	70.5 ± 5.9	52.0 ± 7.7	0.07

*ns* not significative (*P* > 0.1).

## Discussion

In general, parity did not affect ewe-lamb behaviours immediately after birth, as did occur in other previous studies^2,10,20^. Additionally, parity did not influence lambs’ birth weight, different to that published in others studies of the same breed, in which MUL ewes produce heavier lambs than PRI ewes^21^. This is consistent with the difference in BW of PRI and MUL ewes from conception until lambing, indicating that PRI ewes invested proportionally more energy than MUL ewes in the development of their foetus rather than investing in maintaining their growth rate. Although pregnancy is a greater metabolic challenge for PRI than for MUL ewes^13^, it would appear that in these study conditions, PRI ewes prioritized the foetus at the cost of their own growth. The PRI ewes carried a greater foetal burden through gestation than MUL ewes in relation to the maternal BW, which is consistent with lower blood protein concentration in PRI ewes, a consequence of higher protein expenditure for foetal growth. In this experiment, it should also be noted that the level of nutrition did increase from day 100 of gestation until lambing, and thus, was sufficient for PRI foetuses to reach a birth weight within the range of the Corriedale breed^22^. Despite ewes from both groups had access to the same quantity of pasture and supplementation, MUL ewes were heavier during gestation. Thus, food might have had greater impact in PRI than MUL ewes. An alternative interpretation for this result could be that the undernourishment restricted the potential of MUL ewes to produce heavier lambs at birth. In this case, foetuses from MUL ewes may have suffered more with food being restricted during gestation than those from PRI ewes. This also coincides with the tendency of the lambs from MUL ewes to vocalize less at birth than the lambs from PRI ewes, which suggests that the former lambs were less vital and/or had less energy reserves. It should also be considered that this study was performed in a flock in which maternal behaviour score^23^ was considered for many years in selecting the ewes that remain for breeding^7^, probably improving their maternal behaviour.

The present results also provide input on possible differences of the effects of parity on the length of the sensitive period of maternal responsiveness, as PRI and MUL ewes differed in their selective maternal behaviour as early as 2 h after birth. In general, while inexperienced mothers were accepting more of their own lamb than that of the alien, experienced mothers were receptive similarly of both lambs at that time. This indicates that PRI ewes ended their sensitive period of maternal responsiveness earlier than MUL ewes, expanding previous suggestions in sheep^24^ and information available also in goats^25^. Probably the greater incidence of lamb rejection and mortality rates of lambs born from PRI ewes^14,26^ can be explained not only by their less intensive maternal behaviour^20^ but also, by a shorter sensitive period after which they would not recognize any lamb^24^. Additionally, the main lambs’ behaviour and lambs’ BW and body temperature at birth, did not differ between groups, likely due to birth weight and lamb’s body temperature, both of which are related to the lambs’ behaviour around birth in the same Uruguayan extensive sheep production system^27^.

Lambs born from PRI and MUL ewes did not differ in their birth weight, but those born from MUL ewes tended to be heavier than those born from PRI ewes during the lactation period. This might be explained by the greater milk yield produced by MUL ewes, and the tendency to provide access to more solid contents. The greater milk production in MUL than PRI ewes is in agreement with previous reports in sheep^28,29^, and might be related to a better energy balance in the former along the lactation period^30,31^, coinciding with the spring in which ewes accessed better pastures^32^ and received supplementation. On the other hand, ewes’ parity did not influence the behavioural response of their lambs during the short-term maternal separation test, indicating that the lambs remained with similar attachments even at 3 months of age. This result reinforces the initial interpretation indicating that in these conditions, MUL and PRI ewes established similar bond strength. Moreover, it also fits with the interpretation of Freitas-de-Melo et al.^32^, who suggested that the milk yield is not the main factor determining the strength of the ewe-lamb bond during the lactation period, as proposed by Arnold et al.^33^.

Lambs born from MUL ewes had a greater heart rate than those born from PRI during the separation and subsequent reunion of lambs and their mothers. However, it should be considered that the initial heart rate was recorded whilst they remained with other lambs from the flock, but separated from their mother for 30–60 min. Therefore, the general difference in heart rate was a consequence of the general stressful situation, without basal recordings. The difference in heart rate could be considered to be even greater perhaps as lambs reared from MUL ewes tended to be heavier than those reared from PRI ewes during lactation. In this sense, lighter and younger lambs have greater heart rates than heavier and older lambs^34,35^ due to their bigger relative costs of maintenance. Another complementary and non-opposed explanation is that lambs reared by MUL ewes were more stressed by the loss of their mother as she produced more milk, as previously reported in lambs at weaning^8^. On the other hand, during the short-term maternal separation test, all lambs increased their time walking after including the mother in the pen, which indicates that the presence of their mothers on the other side of the fence generated greater anxiety in all the lambs. Therefore, it appears that although the behavioural response between groups did not differ, this would indicate a comparable motivation to get back together with their mothers.

In conclusion, it seems that although PRI ewes had a lower BW and a poor metabolic status throughout the gestation, this difference did not affect the ewe-lamb bond at birth and at 3 months postpartum. At least under nutritionally restricted conditions during gestation, inexperienced mothers appear to have had a shorter sensitivity period of maternal responsiveness than that of experienced mothers.

## Methods

### Location and animal management

All the procedures were approved by the Comisión de Ética en el Uso de Animales, Facultad de Agronomía (Universidad de la República, Uruguay), in accordance with national guidelines and regulations, and with the ARRIVE guidelines. The experiment was carried out at the Estación Experimental Bernardo Rosengurtt, Facultad de Agronomía, Universidad de la República (Cerro Largo, Uruguay; 32° S) using PRI and MUL Corriedale ewes. Oestrous cycles of all the ewes were synchronized with an intravaginal sponge impregnated with medroxyprogesterone acetate (60 mg, Syntex, Buenos Aires, Argentina) during 6 days plus a dose of a PGF2alpha analogue (10 mg, Dinoprost tromethamine, Lutalyse, Pfizer, Kalamazoo, MI, USA) and 200 IU of eCG (Novormon, Syntex, Buenos Aires, Argentina) at sponge withdrawal. Ewes were joined with marking vasectomized rams (1 male:10 ewes ratio). Marks on the rumps of the ewes were recorded twice daily, and marked ewes were inseminated with fresh semen 12 h after oestrus detection (Day 0). Pregnancy status and foetal number were determined on Day 30 with transrectal ultrasound, and only ewes carrying a single foetus were included in the study (14 PRI ewes: 5 carrying female and 9 male foetuses; 21 MUL ewes: 14 carrying female and 7 male foetuses).

From 30 to day 143 of gestation, all ewes remained grazing natural pastures (pasture allowance: 6 to 10 kg of dry matter (DM)/100 kg of body weight (BW)/day; crude protein: 6.4 to 6.7% and metabolizable energy: 2.0 to 2.1 Mcal/kg of dry matter) on three paddocks of approximately 3 ha separated by electric fences and with free access to water. With this pasture allowance, during the aforementioned gestation period, pregnant ewes suffered a restriction up to 30% of their energy requirements and 60% of their protein requirements^32^. From day 100 of gestation until lambing, ewes were supplemented daily with 300 g/animal of rice brain provided collectively (88% DM, 14% crude protein, 9% acid detergent fibre and 24% neutral detergent fibre). Despite of supplementation from day 100 of gestation, at day 143 of gestation, ewes were still in negative protein balance of 12%, but achieved a positive energetic balance of 56%. From day 143 of gestation until parturition, all ewes were kept in a paddock of approximately 2.5 ha, grazing native pasture during daylight. During the night, they were moved to a 40 m × 20 m paddock located close to the former paddock, with artificial dim light to allow observations.

From one week after parturition until the end of the study, all ewes and their lambs grazed in a paddock of approximately 2 ha on native pasture. During this period, all animals were supplemented daily with 300 g/animal of rice brain provided collectively. All ewes were shorn 30 days after lambing.

### Recordings during gestation

Ewes’ body weight (BW) and body condition score (BCS) were recorded in the morning of days 0, 30, 55, 69, 86, 108, 123 and 143 of gestation. Blood samples were collected from all ewes by jugular venipuncture in the morning of days 30, 100 and 143 of gestation. Samples were placed in tubes without anticoagulant and centrifuged for 10 min at 1500 g within 30 min after collection. Serum samples were separated and frozen at  − 20 °C. Glucose, cholesterol, serum total protein and albumin concentrations were measured by colorimetry using commercial kits (Bio-Systems, Barcelona, Spain). All biochemical analyses were assessed using a WienerLab.BT 3000 Plus/CB 350i (Rosario, Argentina) automated chemistry analyser previously used in sheep^32^. Serum globulin concentration was estimated by subtracting the serum albumin concentration from the serum total protein concentration.

On the morning of day 143 of gestation (from 08:00 h to 10:00 h), the following udder measures were recorded: circumference (circumference of the udder near the belly), width (from left insertion of the udder to right insertion) and depth (from insertion of the udder to the inferior extremity). Each semiaxis was calculated, and the udder volume was calculated as in Freitas-de-Melo et al.^32^, assuming that the udder was a semi-sphere: V = 2/3π.R1.R2.R3, where: each R corresponds to each semiaxis (horizontal, depth and width).

### Recordings during the first hours after birth

All ewes were continuously observed during the 24 h period by six trained observers, who remained 5 to 7 m from each ewe when behaviours indicative of the proximity of parturition began (a distance enough to avoid disturbing the normal behaviour of the animals) until lambing occurred. Then, the following data was recorded according to Freitas-de-Melo et al.^32^: duration of the first licking (the total time spent by the ewe licking its lamb for the first time), latency of the lamb from parturition to standing up (the time required by the lamb to maintain an upright position on extended legs for at least 5 continuous seconds), latency from parturition to first suckling (the time taken by the lamb to access its mothers’ teat and suckle for at least 5 continuous seconds), duration of first suckling (total time spent by the lambs sucking for the first time) and number of vocalizations of the mother and the lamb from birth until the first suckling occurred. The frequency of vocalizations of ewes and lambs were calculated as the number of vocalizations/latency from parturition until the first suckling occurred. After the first suckling had ended, the rectal temperature, surface temperatures on the skin of the interscapular zone and of the hips of the lambs, were measured with the aid of a laser thermometer (IR-102 Infrared Thermometer, Super Elec. Equip. Co, China). Thereafter, lambs were tagged and weighed, and their sex registered. Lambing assistance was provided to ten ewes (five MUL and five PRI ewes), as the second stage of parturition (from appearance of foetal front or rear legs to complete lamb expulsion) took more than 30 min with no progress and/or with evidence of meconium in the amniotic fluid^36,37^. Suckling assistance was provided when the latency from parturition to first suckling took more than 2 h (one lamb born from MUL and two born PRI ewes). In these cases the latency to suckle for the first time was considered as 120 min.

A maternal selectivity test was performed approximately 2 h after birth in a 2 m × 1 m pen placed near the pen where the ewes lambed. Each dam was subjected to two successive tests, lasting 3 min each: one with its’ lamb and the other with an alien of similar age and physical appearance (colour and size). The selection and the order of presentation of their own lamb and that of the alien were randomized between ewes, within each group. The number of the following behaviours were counted according to Poindron et al.^38^: (1) low-pitched bleats (number of vocalization emitted with the mouth closed), (2) acceptances at the udder (number of times that the lamb engaged the head in the inguinal region of the ewe, without being rejected), (3) the time near the udder (time spent by the lamb at the udder, with its head engaged in the inguinal region of the ewe during at least 5 continuous seconds), (4) refusals at the udder (number of times that the lamb engaged the head in the inguinal region of the ewe, it was interrupted within less than 5 s by the ewe moving away and showing back leg movement or any aggressive behaviour), (5) aggressive behaviours towards the lamb (head butts and threats), (6) high-pitched bleats (number of vocalizations emitted with mouth open, indicating displeasure). The first three behaviours indicate acceptance of the lamb, whereas the last three indicate rejection. At the end of the test, ewes and their lambs were moved to a larger pen where they remained together with other mothers and lambs from the flock.

### Recordings during lactation

#### Mother-lamb short-term separation test

Three months after lambing, lambs were subjected to a temporal separation test. The test was similar to that described by Barnard et al.^15^ and Freitas-de-Melo et al.^32^, in a pen that was divided into two similar areas of 3.0 m × 2.5 m by a wire mesh plastic fence. A reference line was drawn 80 cm parallel to the fence on the lamb side of the pen. All lambs from each group were separated from their mother 30 to 60 min before testing and placed all together in a pen (6.0 m × 5.0 m) 30 m away from that in which their mothers remained, where they neither can see nor smell each other. Each lamb was introduced separately during 5 min intervals to one area of the testing pen, registering the total time that the lamb spent walking and the number of vocalizations emitted. Thereafter, the mother was introduced on the other side of the fence, and the number of times that the lamb crossed the line, entering to the area (< 80 cm from the fence) was also registered during a 5 min interval. In addition, the time each lamb spent into this area, the total time walking, the number of vocalizations emitted, and the number of attempts to reunite with its’ mother hitting against the fence with any part of the body, were also recorded. Lambs’ heart frequency and rectal temperature were measured immediately before, and after the tests.

#### Milk yield and composition, body weight and body condition score of ewes and their lambs

On days 45 and 91 after lambing, ewes were weighed and their BCS registered, and their lambs also weighed. On day 91 postpartum, all ewes were milked to estimate milk yield according to Freitas-de-Melo et al.^39^. Briefly, lambs were separated from their mothers, receiving 5 IU of oxytocin, and were milked completely. Six hours later, ewes were milked with the same protocol, and daily milk production was calculated based on the 6-h production. Milk fat, protein and lactose concentrations were measured according to ISO 9622.2013 (IDF 141) using a Combi Foss FT + (Foss Electric, HillerØd, Denmark), and a Combi Bentley 2300 (Bentley Instrument USA) equipment. The quantity in grams of milk fat, protein and lactose were calculated considering the concentration (%), the total volume produced per ewe, and the density of ewe milk.

#### Statistical analyses

The experimental design applied was a randomized complete design, where the animal was the experimental unit. Not all the data could be recorded in all the animals, so the exact number of animals is included in the results of each variable. All the variables were tested for normal distribution with the Shapiro–Wilk test. The behaviours and body temperature recorded at birth, and the parameters recorded after including the mother in the short-term maternal separation test were not normally distributed, and thus, were compared between groups with the Mann–Whitney test. The behaviours displayed by each ewe in the selectivity test toward its own lamb or the alien were compared with the Wilcoxon test in each group. The aforementioned variables were analysed using the software Past 2.02, and the remaining data analysed using the SAS University Edition (SAS Institute, Cary, NC, USA). To evaluate the effect of group, the biochemical measurements, BW and BCS variables were analysed separated during gestation period and during postpartum period. The aforementioned variables, heart rate, rectal temperature, the number of vocalizations and the square root of the time walking during the short-term maternal separation test were analysed using a repeated measure analysis. To perform the analyses, a linear mixed model was applied, including the fixed effects of group, time, and the interaction between groups and time, and the random effects of sex and animal The udder volume, milk yield and composition were also analysed using a linear mixed model, including the fixed effects of group, and the ewe as random effect. The least-square means were compared using the pdiff option of SAS. Results were considered significantly different when *p* ≤ 0.05, and a tendency when 0.05 < *p* ≤ 0.1. Data are presented as LSmean ± sem.

## Data Availability

The datasets generated during and/or analysed during the current study are available from the corresponding author on reasonable request.
